# Practical guide to the diagnostics of ruminant gastrointestinal nematodes, liver fluke and lungworm infection: interpretation and usability of results

**DOI:** 10.1186/s13071-023-05680-w

**Published:** 2023-02-08

**Authors:** Gustavo Adolfo Sabatini, Fernando de Almeida Borges, Edwin Claerebout, Leonor Sicalo Gianechini, Johan Höglund, Ray Matthew Kaplan, Welber Daniel Zanetti Lopes, Sian Mitchell, Laura Rinaldi, Georg von Samson-Himmelstjerna, Pedro Steffan, Robert Woodgate

**Affiliations:** 1 Boehringer Ingelheim Animal Health, Ingelheim am Rhein, Germany; 2 Universidade Federal de Mato Grosso do Sul, Campo Grande, Brazil; 3 Ghent University, Ghent, Belgium; 4 University of Georgia, Athens, USA; 5 Swedish University of Agricultural Sciences, Uppsala, Sweden; 6 St. George’s University, St. George’s, West Indies Grenada; 7 Universidade Federal de Goias, Goiania, Brazil; 8 The former Animal and Plant Health Agency (APHA), Perth, UK; 9 University of Naples Federico II, Naples, Italy; 10 Freie Universität Berlin, Berlin, Germany; 11 Fiel & Steffan Consultores Asociados, Tandil, Argentina; 12 University of Adelaide, Roseworthy, Australia

**Keywords:** Ruminants, Parasite, Diagnostics, Fecal egg count, Coproculture, FAMACHA^®^, Plasma pepsinogen, ELISA-*Ostertagia*, ELISA-*Fasciola*, Baermann and ELISA-Lungworm

## Abstract

The diagnostics of ruminant parasites remains one of the cornerstones for parasite control best practices. Field veterinarians have several techniques at their disposal (fecal egg count, coproculture, FAMACHA®, plasma pepsinogen, ELISA-*Ostertagia*, ELISA-*Fasciola*, Baermann and ELISA-Lungworm) for the identification and/or quantification of gastrointestinal nematodes, lungworms and liver fluke infecting small ruminants and cattle. Each of these diagnostic tools has its own strengths and weaknesses and is more appropriate for a specific production operation and/or age of the animal (young and adults). This review focuses on the usability and interpretation of the results of these diagnostic tools. The most advanced technical information on sampling, storage, advantages and limitations of each tool for different types of production operations and animal categories is provided.

**Graphical abstract:**

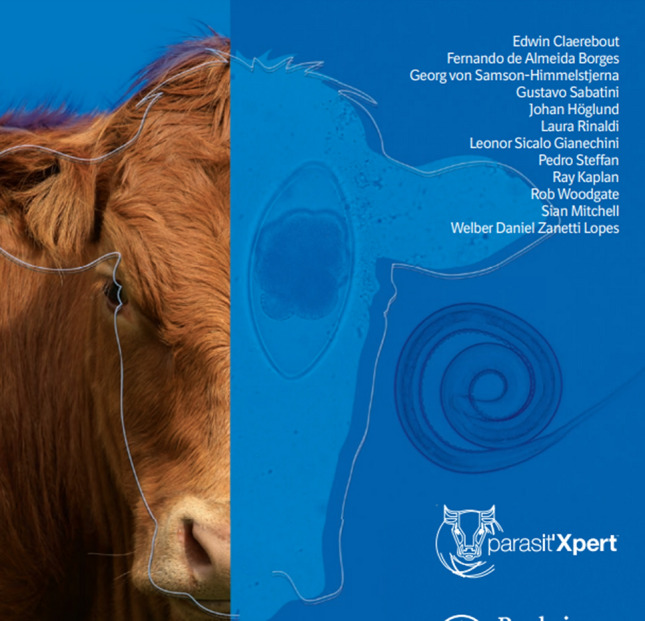

## Background

Historically, many deworming programs were characterized by their calendar-based whole-herd/flock blanket treatments. Also, in the past, particularly in some areas where climatic conditions favor the development of pre-parasitic stages in the environment, animals were administered anthelmintics on a 2-weekly or monthly basis [1]. This approach has led to the development of resistance to most anthelmintics currently available on the market. Resistance issues were until recently solved by treating animals with new products based on new active pharmaceutical ingredients that had innovative modes of action [2]. However, although new anthelmintic molecules, such as monepantel and derquantel, were developed in the last decade, no new endectocide class anthelmintic has been developed since the early 1980s, when Ivomec® (ivermectin, the first macrocyclic lactone) was launched. Due to more stringent food safety and ecotoxicity regulations, the development of new products has become even more complex, resulting in much higher costs and longer times before the product is commercially available. Therefore, it is unlikely that new innovative products will arrive on the market fast enough to outpace the development of resistance. Consequently, chemical treatments should increasingly be based on need, preceded by diagnostic results, and adapted to local conditions on the farm [3].

This critical situation requires coordinated efforts from the animal health industry, scientific community, veterinary practitioners, policy-makers, producers and other stakeholders and demands a complete paradigm shift in the approach to parasite control. It is imperative to move away from the exclusively chemical approach and instead move toward the implementation of best practices for parasite control. There is no longer a “one size fits all” solution; multiple drug resistance has forced a return to the basics of parasitology to determine the most effective and sustainable parasite control programmes [2].

Diagnostics play an important role in reaching this objective; however, the success of a parasite control program is also linked to additional factors, such as parasitological history and husbandry practices of the farm. Monitoring epidemiology and weather conditions is also important, and when such data are not available at the farm level, regional data could be an option.

The purpose of this guidance is to provide practical advice at the farm level on the usability and interpretation of the results of ruminant internal parasite diagnostic tools. This document focuses on the techniques currently available for producers/veterinarians and contains a summary of the most advanced scientific information on this topic. The result is a compilation of practical instructions on “why” and “when” to use each of the available tools and, ultimately, on how to interpret the results. Several techniques currently available only in the scientific realm, for example, quantitative-PCR, loop mediated isothermal amplification (LAMP), droplet digital PCR (ddPCR) and next-generation sequencing-nemabiome barcoding, do not fall within the scope of this document.

## Fecal nematode egg count

Several techniques are available to perform fecal egg counts (FEC) in cattle and small ruminants. In general, an FEC consists of weighing a sample of freshly collected feces, homogenizing the sample with a flotation solution and then filtering or centrifuging the fecal slurry to remove large debris particles; the flotation principle separates the eggs for identification and quantification using a microscope. FEC can be used for the diagnosis of individuals or groups (pooled or composite sampling).

The McMaster technique [4] is the most widely used method for diagnosing gastrointestinal nematode (GIN) infection because it is easy and inexpensive to run and does not require sophisticated laboratory equipment [5]. Another well-known technique is the modified Wisconsin protocol [6]. In addition, several refinements of FEC methods have been developed in recent years, such as the Mini-FLOTAC [7] and FECPAK [8]. More recently, automated FEC techniques that use artificial intelligence and machine learning for automated recognition and counting of helminth eggs have been developed; some of these may become commercially available in the near future.

### Important things to know about FEC

**Fig. 1:**
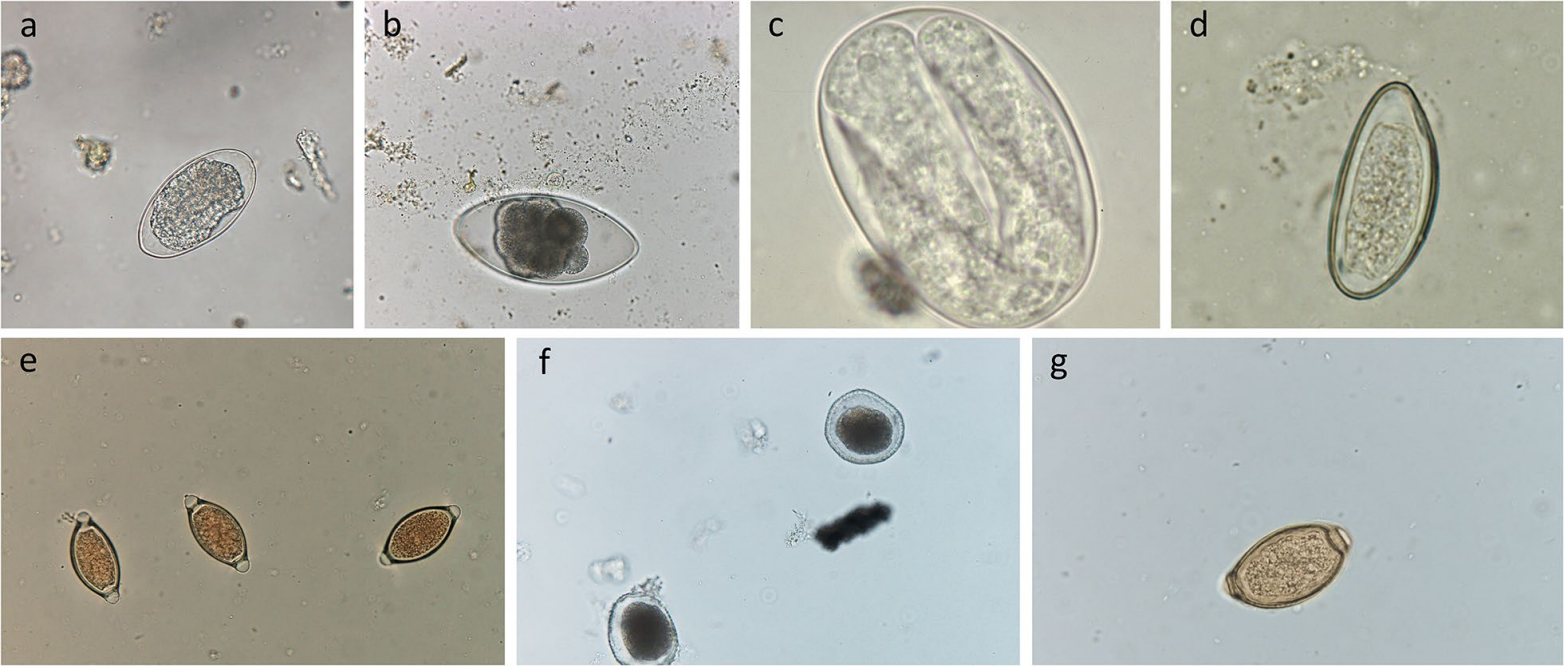
Eggs of gastrointestinal nematodes of livestock that are commonly seen in fecal samples. **a**  Strongyle-type egg, **b** *Nematodirus* spp. egg, **c** Strongyloides spp. egg, **d** *Skrjabinema* spp. egg, **e** *Trichuris* spp egg, **f** *Toxocara* spp. egg, **g** *Capillaria* spp. egg

- Timing of sampling matters. The FEC usually decreases with increasing host age due to immunity; however, it can also increase in adults depending on breeding status and/or season [9].
- Number of sampled animals must be adequate. Sampled animals should be in the same category (young, adults, heifers, etc.) and maintained in the same pasture under the same management activities. The higher the number of sampled animals, the better.
  - Sheep operations. A pooled sample of 10 sheep allows for a reliable estimate of the mean FEC in most flocks, provided that an equal amount of feces is collected from each animal and the fecal samples are thoroughly mixed in the flotation fluid [10].
  - Cattle farms: At least 10 animals (or 10% of the group) by category (young, adults, etc.) should be sampled [11].
- Correct storage and shipment of fecal samples. For practical reasons, fecal material requires proper storage prior to coprological examination. Inadequate storage conditions can cause a reduction in egg numbers. An artefactual reduction in FEC occurs primarily due to either the hatching of eggs or biological degradation. If using bags for collection, air must be squeezed out before they are sealed; if using pots, the pots should be filled to the brim to exclude air. Samples should be kept cool (approx. 4 °C) and analyzed within a few days of collection. The recommendation is to place bags or containers with feces inside a cooler containing freezer packs while avoiding direct contact of the bags or containers with the freezer packs (e.g., by using a thick layer of newspaper). If a coproculture is to be performed, avoid storing the samples in the refrigerator longer than overnight.
- Variation in methodologies impacts results. There are many technical sources of variability in FEC results, including pre-analytical factors (such as collection, labeling and storage of fecal samples) and analytical factors (such as volume/weight of fecal samples, filtration, homogenization and flotation solutions). The most important factor is consistency of the FEC protocol used over time. Using the same laboratory for examination of different samples (and obviously the same FEC methodology) enables the comparison of results over time, allowing a track record of the parasitological status of each of the herds under the care of the veterinary practitioner to be recorded. More information on the impact of the issues/results related to different FEC methods is available from Nielsen [12].
- Eggs from different species cannot always be differentiated. The most important GIN of livestock are taxonomically included in the superfamilies Trichostrongyloidea and Strongyloidea; therefore, the results of FEC techniques are given in numbers of trichostrongyle or strongyle eggs per gram (EPG) of feces. Morphometry of the eggs of different GINs overlaps considerably across genera and species, preventing genus- or species-level identification. The exceptions are illustrated in Fig. 1: the genera *Nematodirus*, *Trichuris*, *Capillaria* and *Strongyloides* found in both cattle and small ruminants; *Skrjabinema* in small ruminants and *Toxocara* in cattle.
- Different helminths produce different quantities of eggs per day. In regard to daily egg production, some parasites are more prolific than others. If the main genus infecting cattle is *Ostertagia* (not a very prolific helminth), a significant productivity impact can be explained by a low to moderate egg count. Daily egg production by different species of GIN is shown in Table 1.
- Consistency of the feces also influences results: Anyone who has been both on dairy farms and beef operations has noted the difference in the consistency of feces. Dairy cows normally have more liquid feces than beef animals. Animals suffering from specific diseases can have diarrhoea. The amount of water in the sample should be considered when drawing conclusions because eggs might be diluted in a watery sample, and various adjustment factors have been proposed for sheep [13].
- Interpretation of results is not straightforward. FEC results should be interpreted with caution because several factors can influence the results. One such factor is parasite pathogenicity: low FEC results originating from more harmful species (such as *Ostertagia* in cattle) could explain significant productivity losses.

**Table 1:** Estimation of egg production per parasite per day

Helminth	Daily egg production
*Haemonchus*	5000–10,000
*Trichostrongylus*	100–200
*Cooperia* *oncophora*	1100–4400
*Ostertagia* *ostertagi*	200–350
*Stongyloides* *papillosus*	Approx. 3.000
*Nematodirus*	50–100
*Oesophagostomum,* *Chabertia*	Approx. 3000
*Toxocara*	Approx. 200.000
*Fasciola* *hepatica*	2000–8000

References: [16–18]

The answers to the questions below will provide valuable information for interpretation of the results:

- Date of sampling
  - Animal’s parasite burden (and parasite species composition) varies throughout the year. Therefore, it is important to get to know the epidemiology of the most important parasites in your region.
- What animal category (calves, cows, bulls, lambs, ewes etc.)/cattle genotype (*Bos indicus* vs.* Bos taurus*) was sampled?
  - As mentioned above, when animals (either cattle or sheep) grow older, they develop immunity that reduces worm fecundity. Consequently, egg count becomes a less reliable indicator of the worm burden size. Small ruminant adult females become less resistant to parasites close to parturition.
- When was the last antiparasitic treatment performed and what product was used?
  - Active pharmaceutical ingredients present different efficacy profiles, and some are more efficient against specific parasites than others. For example, *Cooperia* is the dose-limiting parasite for macrocyclic lactones, and benzimidazoles present variable efficacy against *Ostertagia*-inhibited larvae.
  - Depending on the formulation, a parasiticide will provide a shorter or longer persistent activity, and some do not even provide persistent activity.
  - Always check the product label to understand its indications and duration of antiparasitic activity.
- Type of production operation
  - Feces from dairy animals are usually more liquid than feces from beef cattle.
- Stocking rate
  - The higher the stocking rate is, the higher the potential parasitological pressure [14]. More animals per hectare means that animals will feed close to dung pats, increasing the chance of infective parasite larvae ingestion.
- Nutritional status and food availability.
  - Animals in good nutritional condition with a proper diet are more resistant/resilient to parasite infection [15].
- Other information that might be important for the interpretation of the results refers to the level of pasture infectivity and the weather in the last few weeks of sampling.

### FEC: focus on cattle

The interpretation of FEC from cattle is not straightforward [19]. A bovine produces approximately 10% of its weight in feces per day; therefore, a 500-kg cow will excrete approximately 50 kg of feces per day. Usually, a sample of approximately 20–40 g of feces is collected to perform an FEC from which a smaller proportion is analyzed (usually 4 g). This means that the result of the FEC will be based on a sample of just 0.008% of the total amount of feces produced by that animal on that day.

The most important drawback of the FEC is that, depending on the GIN genera/species, there may not be a consistent relationship between it and worm burden (with the exception of young animals at the beginning of the grazing season). However, FEC remains a prognostic tool for measuring/estimating how contaminated the pasture becomes with parasite eggs.

A high FEC (> 200 eggs per gram [EPG] in Europe and > 500 EPG in South America) means a high chance of an important parasite burden. However, a low FEC (< 50–100 EPG) does not necessarily mean that the animal will not benefit from anthelmintic treatment. For example, a low FEC could be the result of poor or untimely sampling, or it could reflect a host reaction that is shifting energy that should be used for weight gain or milk production to a very demanding immune system to maintain parasitism at a low level.

As mentioned, the age of the animals, type of production operation, nutrition status and breed (species) should also be taken into consideration since European and Indian breeds differ in susceptibility to parasites [20, 21]. Another aspect of the FEC results that could influence their interpretation is helminth pathogenicity. As an example, *Ostertagia* is more pathogenic but less fecund than *Cooperia*, and *Haemonchus* is both pathogenic and fecund. A last interesting fact about cattle FEC is that *Ostertagia* egg production per helminth decreases when the worm population surges in the host abomasum.

#### In which situations can FEC add value to cattle operations?

- Confirmation of GIN parasitism and differential diagnosis from other causes of diarrhoea and ill-thrift [22].
- Screening for the most efficacious anthelmintic (or a treatment efficacy check). In this context, a FEC reduction test (FECRT) is recommended to determine the susceptibility/resistance status of a specific farm’s GIN population to different anthelmintics [11]. In this test, feces from a group of animals are collected before and after treatment for FEC determination. The pre- and post-treatment FECs are used to calculate the product efficacy. For more details on how to proceed with an FECRT, the reader is referred to the COMBAR guideline [23]. Due to different persistent efficacy profiles, the time for fecal collection after treatment varies according to the drug class used (see Table 2). In a modification of the FECRT, pre-dose FECs are not performed, and the results are based on the percentage reduction in mean FEC in the treatment groups compared to the non-treated controls.
- Post-drenching check. This is a less structured approach to check product efficacy. Instead of collecting samples before and after treatment, feces are collected only after treatment (Table 2) and pooled for the FEC. This alternative is not as reliable as the formal FECRT; on the other hand, it is less costly and less time-consuming.
- To check egg secretion of newly purchased animals before they are released to pasture with the stationary herd.
- Pasture contamination measurement (specifically in western Europe). FECs may be useful during the early part of the grazing season, as the number of worm eggs shed during this period (partly) determines the number of infective larvae on the pasture in the second half of the grazing season. If, approximately 2 months after turnout, the geometric mean FEC (at least 20 animals should be sampled) is > 200 EPG, animals should be immediately treated to avoid outbreaks of clinical parasitic gastroenteritis (PGE) [5]. It is worth mentioning that when the geometric mean FEC < 200 EPG, the chance of a clinical outbreak occurring falls to 30%. It is important to highlight that this threshold (200 EPG) relates to clinical parasitosis, but it is mostly important to avoid losses due to subclinical parasitosis [24].
- Targeted treatment (TT). The low FEC results usually found in samples from animals raised in the Northern Hemisphere (and dairy cattle from the Southern Hemisphere) have prevented any attempt to generate a successful FEC threshold for subclinical PGE treatment. However, in tropical and subtropical areas, where the parasitic pressure is much higher, FEC results might be more valuable. Based on a recent paper [13], it is possible to recommend treatment thresholds for Brazil and perhaps also for properties in other countries at the same latitude, comparable production systems (extensive grazing conditions) and a similar mix of helminth infections (60–75% *Cooperia*; 15–25% *Haemonchus*; 10–15% *Oesophagostomum*; < 5% *Trichostrongylus*). If a minimum of 30% of the animals in a herd (independent of the category [nursing or weaned calves, heifers, adults, etc.]) present approximately 250 EPG, treatment of the whole herd is justified to avoid losses due to subclinical parasitosis.

**Table 2:** Time period for post-treatment sampling according to different chemical compounds

Tested drug	Post-treatment sampling (*n* days post-treatment)
Levamisole	7–10
Benzimidazoles	10–14
Ivermectin and other macrocyclic lactones	14–17
Moxidectin	17–21
When testing ≥ 2 drugs in same herd/flock	14

It is important, of course, to acknowledge that regardless of the results (low or high), FEC results might open a window of opportunity for veterinary practitioners to engage with producers on parasitology. However, if FECs are being used as a basis for advice regarding control options, then additional parameters must also be considered (such as coproculture results, parasitological history and husbandry practices of the farm, epidemiology, weight gain, milk production and weather conditions); otherwise, there is a risk of inappropriate actions being taken.

### FEC: focus on sheep

As compared to cattle, the benefits of FEC are somewhat clearer in sheep, although, as in cattle, FECs should be viewed as additional diagnostic information to be considered alongside history and clinical signs. Careful interpretation of the results is particularly important when the FEC is low.

Despite the differences in fecundity and pathogenicity among sheep GIN, particularly in young animals, FECs are better correlated with worm burdens of *Haemonchus* *contortus* and *Trichostrongylus* spp. Another interesting observation is that the fecundity of adult female *Teladorsagia* is inversely density dependent; in other words, egg production per worm is higher when the number of worms in the gut is low [25].

In outbreaks of acute GIN infection, the initial mean FEC in a group of animals may be low because the infection has not yet become patent. Notably, prepatent *Nematodirus* *battus* infection in lambs and prepatent *H.* *contortus* infections in sheep of any age can be associated with severe disease and even death. When interpreting FEC results, it should always be remembered that the eggs were produced by worms picked up by the sheep 3 or 4 weeks earlier. FECs provide no information on the number of juvenile and premature nematodes present in the animal at the time of sampling.

In sheep, FEC results are usefully combined with the results of coproculture and both can be used to guide treatment, especially when *H. contortus* is present.

In Australia, several drench decision guides across multiple geographic regions have been developed to assist farmers in making intervention decisions based on FECs [26]. Table 3 shows an example of a drench decision matrix based on current nutrition levels, animal condition (measure of previous nutrition) and dominant worm species in a summer rainfall region. These FEC thresholds are valuable when the animals are predominantly infected by *Haemonchus* and *Trichostrongylus*. Currently, it is not possible to generate FEC thresholds for other species of helminths; the variability in egg output and worm pathogenicity among different worm species has prevented this goal from being achieved. It should be noted that these figures are only validated for Australia.

**Table 3:** Fecal egg count/coproculture results: guidelines for flock treatment

Animal condition	Pasture quality/quantity		
	Poor	OK	Good
*Barber’s pole worm (Haemonchus contortus) > 60% of culture*			
Poor	600	800	1000
OK	800	1000	1100
Good	1000	1100	1200
*Scour worms *(*Trichostrongylus spp.*)			
Poor	300	400	500
OK	400	500	600
Good	500	600	700

Instructions on how to use the table: First, check the animal’s condition (poor, OK or good), then check the pasture for “quality/quantity (poor, OK or good).” The value in the cell at the intersection of the animal condition and pasture quality/quantity assessment is the eggs per gram (EPG) threshold for treatment when the coproculture results indicate > 60% of the parasites as *Haemonchus* or the predominant species is a *Trichostrongylus* species

A guide for the interpretation of FEC results is also available for the UK and Ireland [27]. Those figures are presented in Tables 4 and 5 and should be used together with other factors (epidemiology, history of antiparasitic use, farm husbandry practices and weather information) to provide a more holistic recommendation of when and how (e.g., targeted selective treatment [TST], TT) animals should be treated.

**Table 4:** Guide to the interpretation of individual fecal egg counts in lambs

Worm egg count (EPG)	Comment	Action
50–350	Light infection	Treatment not necessary
400–600	Moderate infection	Anthelmintic treatment may be necessary
650–1000+	Heavy infection	Anthelmintic treatment necessary

Reference: [27]

**Table 5:** Guide to interpretation of pooled (or composite) fecal egg counts in lambs

Worm egg count (EPG)	Comment	Action
< 200	Low egg count	Treatment probably not justified. Continue monitoring
200–500	Some clinical disease could be present	Anthelmintic treatment should be beneficial in individuals
500–1000 eggs	Clinical disease likely in a large proportion of the group	Anthelmintic treatment necessary to a large proportion of the group
1000+ eggs	Clinical disease likely in the whole group with some individuals heavily infected	Anthelmintic treatment necessary to a large proportion of the group

Reference: [27]

#### In which situations can FEC add value to sheep operations?

- Guiding decisions about the need for treatment (including strategic reasons).
- Confirmation of parasitism by GIN and differential diagnosis from other causes of diarrhoea and ill-thrift [22].
- Screening for the most efficacious anthelmintic (or a treatment efficacy check): for details, see point ‘Screening for the most efficacious anthelmintic (or a treatment efficacy check)’ in section In which situations can FEC add value to cattle operations?. For more information on how to proceed with a FECRT in small ruminants, check the COMBAR guideline [28].
- Post-drenching check. For details, see point 'Post drenching check’ in section In which situations can FEC add value to cattle operations?.
- Targeted treatment or TST, as previously mentioned. For details, see section FEC: focus on sheep.
- Assessment of pasture contamination by the free-living pasture stages of the key GIN parasites.
- Identification of animals with low trichostrongylid egg counts to be used as target phenotypes in sheep breeding programs.

### FEC techniques

#### McMaster technique

The McMaster method, developed at the McMaster laboratory of the University of Sydney, is the most universally utilized FEC technique in veterinary parasitology and is advocated by the World Association for the Advancement of Veterinary Parasitology for evaluating the efficacy of anthelmintic drugs in ruminants [29]. Since its detection limit is relatively poor for certain applications, other techniques have been developed, such as the Wisconsin protocol, the improved McMaster technique [30], Mini-FLOTAC and FECPAK, with the last two applications being the most widely used. However, all FEC techniques are based on the principle that feces are mixed with a flotation medium and then the eggs are counted in a different type of counting chamber.

#### Modified Wisconsin technique

The major difference between the modified Wisconsin protocol and the McMaster technique is related to the centrifugation steps. A comparison of several FEC techniques demonstrated that the centrifugation step allowed the most consistent recovery of more eggs from bovine feces than other methods [31].

As with the McMaster protocol, there are several variations of the modified Wisconsin technique used by different laboratories [32].

#### FLOTAC and mini-FLOTAC

The search for methods with higher sensitivity and accuracy led to the development of a multivalent technique [33], known as FLOTAC, for qualitative and quantitative copromicroscopic diagnosis of patent endoparasite infections in animals and humans. FLOTAC is a sensitive test that allows the quantification of 1 EPG of feces. It also allows the diagnosis of lungworm larvae (*Dictyocaulus* spp.) and trematode eggs (*Fasciola* *hepatica*) depending on the type of floatation medium. The advantages of the FLOTAC technique over the McMaster method were demonstrated in a survey of anthelmintic resistance in cattle because it allowed the inclusion of animals with an FEC of < 50 EPG of feces [34]. However, FLOTAC is more time-consuming than the McMaster technique and requires a centrifuge for the plates. These factors were taken into consideration with the development of a more convenient technique, the mini-FLOTAC [7]. The mini-FLOTAC does not require centrifugation and has good sensitivity, allowing the detection of 5 EPGs.

#### FECPAK

The FECPAK method is based on a modification of the McMaster technique and has a minimum detection limit of 30–35 EPG of feces [35]. The original FECPAK method was developed in New Zealand to provide a simple on-farm method for FEC estimation. The updated FECPAK^G2^ method uses a flotation–dilution approach similar to the McMaster technique but involves capturing digital images of samples without the use of a microscope. The digital images of samples are then stored and can be assessed by trained technicians for the identification and counting of nematode eggs [36]. Each digital image remains available for reference and auditing purposes. Setting up the FECPAK^G2^ test does not require specialized laboratory equipment or technical skills, and preparation can be done easily in the field by a lay operator.

#### Summary of techniques for FEC

All of the above-mentioned techniques have their own specific value and can be used in all the situations described above. However, it is worth noting that the lower the detection limit and higher the accuracy and the precision, the better the technique’s fit for a FECRT. In situations where the parasitological pressure is not high, and FEC results are expected to be low, techniques with a low detection limit are preferred. If the demand for the FEC results is urgent, FECPACK is the only pen-side diagnostic tool currently available, and it allows an on-site and immediate discussion about the results with the producer. As a consequence, FECPACK’s cost is generally higher than the other techniques.

Table 6 summarizes the characteristics of the FEC techniques mentioned in this article.

**Table 6:** Characteristics and main limitations of different copromicroscopic techniques used for the diagnosis of helminth infection in ruminants

FEC technique	Diagnostic performance			Technical performance			Comment
	Detection limit	Accuracy	Precision	Cost	Processing time^b^	Equipment needs	
McMaster	Medium^a^	Low	Low	Inexpensive	Medium	Basic laboratory equipment	Most common technique across the globe
Modified Improved McMaster	Medium	Low	Low	Low	Medium	Fully equipped laboratory	
Modified Wisconsin	Very Low	Low	Very low	Low	Long	Fully equipped laboratory	Lack of precision due to the lack of a grid on the coverslip
Mini-FLOTAC	Low	High	High	Low	Very long	Basic laboratory equipment	Allows detection of GIN, lungworm larvae and trematodes
FLOTAC	Very Low	Very high	Very high	Low	Very long	Fully equipped laboratory	Requires centrifugation steps with two different rotors
FECPAK	Medium	Low	Low	Expensive	Long	All equipment is provided by the manufacturer	It is a pen-sided tool. Utility is limited to gastrointestinal strongyles

*GIN* Gastrointestinal nematodes

^a^Detection limit of McMaster. The efficacy of the modified McMaster can be increased by changing the fecal/fluid ratio and/or reading several chambers

^b^Medium: 6 min/sample; Long: 13 min/sample [37]

## Coproculture

Unlike the eggs of *Trichuris* spp., *Strongyloides* spp., *Capillaria* spp., *Nematodirus* spp., *Toxocara* spp. and *Skrjabinema* spp., which are easily identified based on their morphology, the eggs of most strongyle genera (*Haemonchus*, *Ostertagia*, *Trichostrongylus*, *Cooperia* and *Oesophagostomum*) are morphologically similar [16]. For this reason, the best way to interpret the results of fecal examination is by associating strongyle FECs with the identification of third-stage (L3) larvae recovered from fecal cultures to determine the proportions of each nematode genus present based on the number of eggs shed [38]. Detailed descriptions of the differentiation of the infective larvae of nematode parasites of sheep and cattle are available in [39].

The usual recommendation is that 100 L3 strongyle larvae are identified, with the results expressed as a percentage. A frequent mistake is the inclusion of *Strongyloides* *papillosus* larvae in the results. The level of *S.* *papillosus* infection must be evaluated during the FEC because it is possible to differentiate its small embryonated egg from the morulated strongyle eggs. It is very important to consider that *S.* *papillosus* can also develop a generation of free-living adults that rapidly produce eggs, resulting in infective larvae in fecal cultures. For this reason, even cultures initially containing a small number of *S.* *papillosus* eggs may end up with large numbers of *S.* *papillosus* L3 larvae. Therefore, *S.* *papillosus* should not be included in the percentage of nematode larvae identified in fecal cultures [16]. Similarly, *Nematodirus* eggs, which are larger and darker than other strongyle eggs, can be easily enumerated during the FEC, as can the barrel-shaped, thick-shelled *Trichuris* eggs and the rarely occurring round, thick-shelled *Toxocara* eggs. In conclusion, only the percentage of strongyle larvae should appear in the results of fecal cultures.

It takes an additional 7–14 days to cultivate the larvae, and it is worth mentioning that eggs from different helminths do not hatch and/or development equally to the L3 larval stage because storage conditions of the feces and the temperature at which the test is performed may favor one genus over another [40]. It is therefore safer to use the larvae culture results as a general indication of the worm population present, rather than as a precise determination of the proportion of FEC contributed by each genus [41].

### Coproculture in cattle

Since no subclinical thresholds have been defined for the percentage of a helminth genus to drive treatment decisions, a coproculture provide little help in determining whether or not a treatment is necessary. However, coprocultures are useful to determine which species are driving resistance in a property after a FECRT has shown poor results for a specific product and to understand the epidemiology of the parasites.

### Coproculture in sheep

In contrast to cattle, coprocultures are commonly used to drive drench decisions in sheep operations, primarily because the percentage of *Haemonchus* and *Trichostrongylus* in the total worm population plays a decisive role in deciding whether treatment should occur (see Tables 3, 4, 5).

Fluoroscein-labeled peanut agglutinin is also a useful and cheaper test than coproculture for differentiating *Haemonchus* eggs in FECs [42].

## FAMACHA®

The FAMACHA® scoring test was developed as a TST indicator for sheep but it has also been proven useful for testing in goats [43–45]. The prerequisite for successful use of this tool in both sheep and goats is the presence of *H.* *contortus* as the major parasite among the helminth population.

Since *H.* *contortus* is hematophagous, it is possible to use the color of the mucous membranes and red blood cell values (packed cell volume and hematocrit) as signs of parasitosis. The FAMACHA® system consists of a color chart that is used as an indicator of which individuals in a flock should be selectively treated for haemonchosis [46]. The color of the mucous membranes of all sheep in a flock are regularly checked against the FAMACHA® chart, and only those sheep with pale membranes are treated with an anthelmintic. The rationale behind this selective treatment is that it enables clinically affected animals to be identified and treated but also ensures that those not requiring treatment will continue to contaminate the pasture with nematode eggs, thus potentially generating refugia for maintaining the genetic diversity of the nematode, thereby slowing/delaying the development of anthelmintic resistance [47].

FAMACHA® should not be used as a selective criterion in the diagnosis of non-hematophagous parasites [46]. In contrast, the diarrhoea score and body condition score, as well as declines in productivity (weight gain and milk production), FECs and other TST indicators [48], can be used to diagnose both hematophagous and non-hematophagous parasites [49, 50].

When the FAMACHA® chart is used, the frequency of treatment with chemicals can be greatly reduced, on average, by > 50% [46], thereby slowing the development of resistance. However, there are questions regarding its impact on productivity. Most published research on this topic indicates no negative effect [51–54], but authors have pointed to potential losses [46, 55], mainly when FAMACHA© is used in lambs [56, 57]. The FAMACHA® system is considered to be one of the best TST criteria in ewes [51, 52, 58]. However, even when *Haemonchus* is the major parasite, it is not recommended to use the FAMACHA® system as an exclusive criterion for TST in growing lambs. The productive criterion of weight gain in lambs can be effectively used in TST for the control of GIN without productive losses, regardless of any association with the FAMACHA® system [55, 56]. Additionally, it is known that the presence of *Fasciola* and/or *Eimeria* can compromise the success of FAMACHA® implementation [59].

## Enzyme-linked immunosorbent assay–*Ostertagia*

The enzyme-linked immunosorbent assay (ELISA) is an immunoassay that relies on the detection of host antibodies against *Ostertagia* *ostertagi* as an indicator of infection. The ELISA—*Ostertagia* system was initially developed for the analysis of individual serum samples. However, it was further developed and evaluated for application to individual and bulk milk analysis. Despite the fact that the bulk milk ELISA reflects past exposure to the parasite, it is an interesting alternative for monitoring *O.* *ostertagi* infection status in dairy herds, as it allows a rapid and moderately inexpensive diagnostic of parasitism at the herd level [60].

Bulk milk ELISA results can provide timely information on parasite exposure status within the larger picture of a herd health monitoring program. Monitoring on a regular basis (approx. 4 times/year in the southern hemisphere and once per year in a setting with a summer grazing period and a winter housing period) may demonstrate trends in parasite-specific antibody levels and seasonal variations in disease status. The results from bulk milk ELISA for *O.* *ostertagi* are effective in determining production-based thresholds since they provide a useful indicator of subclinical infections and the relative infection status of a herd [61, 62].

A commercial ELISA kit for detecting antibodies to *O.* *ostertagi* in milk samples is available from Indical Bioscience (Leipzig, Germany). Antibody levels are expressed as the optical density ratio (ODR). From an economic perspective, there is an important relationship between bulk tank milk ODR and milk production: the higher the ODR, the lower the herd’s milk production [63]. Anti-*O.* *ostertagi* antibodies in milk are useful indicators in the evaluation of parasite exposure and potential production losses and to inform control plans and treatment decisions [64–66].

**Fig. 2:**
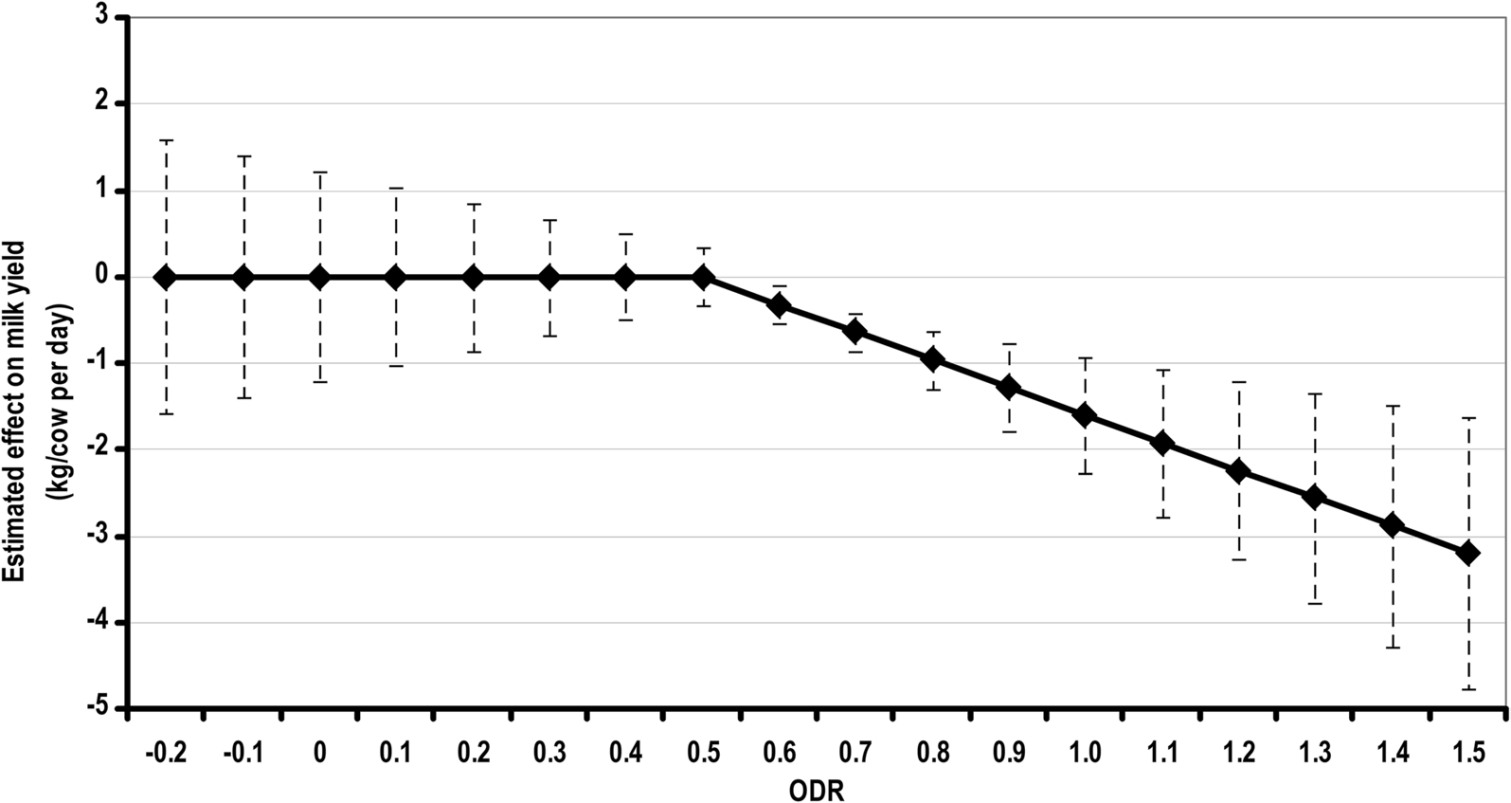
Guide to interpretation of bulk tank milk *Ostertagia* *ostertagi* enzyme-linked immunosorbent assay titers (ODR) in relation to potential impact on individual daily milk yield in dairy herds. To assess the importance of the infection in a specific herd, the herd’s bulk tank milk ODR should be plotted on the line. The probable effect of this level of infestation pressure on the herd average milk yield can be read on the *Y*-axis. ODR, Optical density ratio

A chart was created to aid the user of the kit in the interpretation of the bulk tank milk test results (Fig. 2); results > 0.5 or 0.8 ODR (depending on the geographical region) are associated with an increased risk of production losses due to GIN and therefore may result in an increased milk yield after treatment. However, on some farms with high ODRs, no treatment effect is seen. It should be noted that this threshold has been validated only for some European countries. Similar to many other diagnostic tests, *O.* *ostertagi* antibody titers in bulk milk should not be the sole determinant in the decision-making process regarding estimated losses and potential response to treatment.

## Plasma pepsinogen

The concentration/levels of pepsinogen in blood plasma/serum are related to the extent of abomasal damage caused by parasites such as *O.* *ostertagi*. In the first grazing season, high pepsinogen values in cattle correlate with the occurrence of parasitic gastroenteritis.

When clinical ostertagiosis is suspected, plasma pepsinogen levels provide the means for a “quick” diagnosis on a herd level. Additionally, this technique has been proven to be a useful monitoring tool when used in first-season grazing calves at housing to evaluate parasite exposure in the past grazing season. Together with the farm’s management history (e.g. duration of the grazing season, antiparasitic treatment history), the results allow a discussion on the parasite control activities for the following year. Several authors have published studies on the relationship of pepsinogen levels with worm burden, pasture infectivity, chemoprophylaxis and weight gains [67–71], and the value of this indicator for monitoring purposes has also been reviewed [72, 73].

The main drawbacks of plasma pepsinogen measurement are the lack of a standardized method (comparing results from different laboratories can be challenging) and the requirement for invasive blood sampling.

**Fig. 3:**
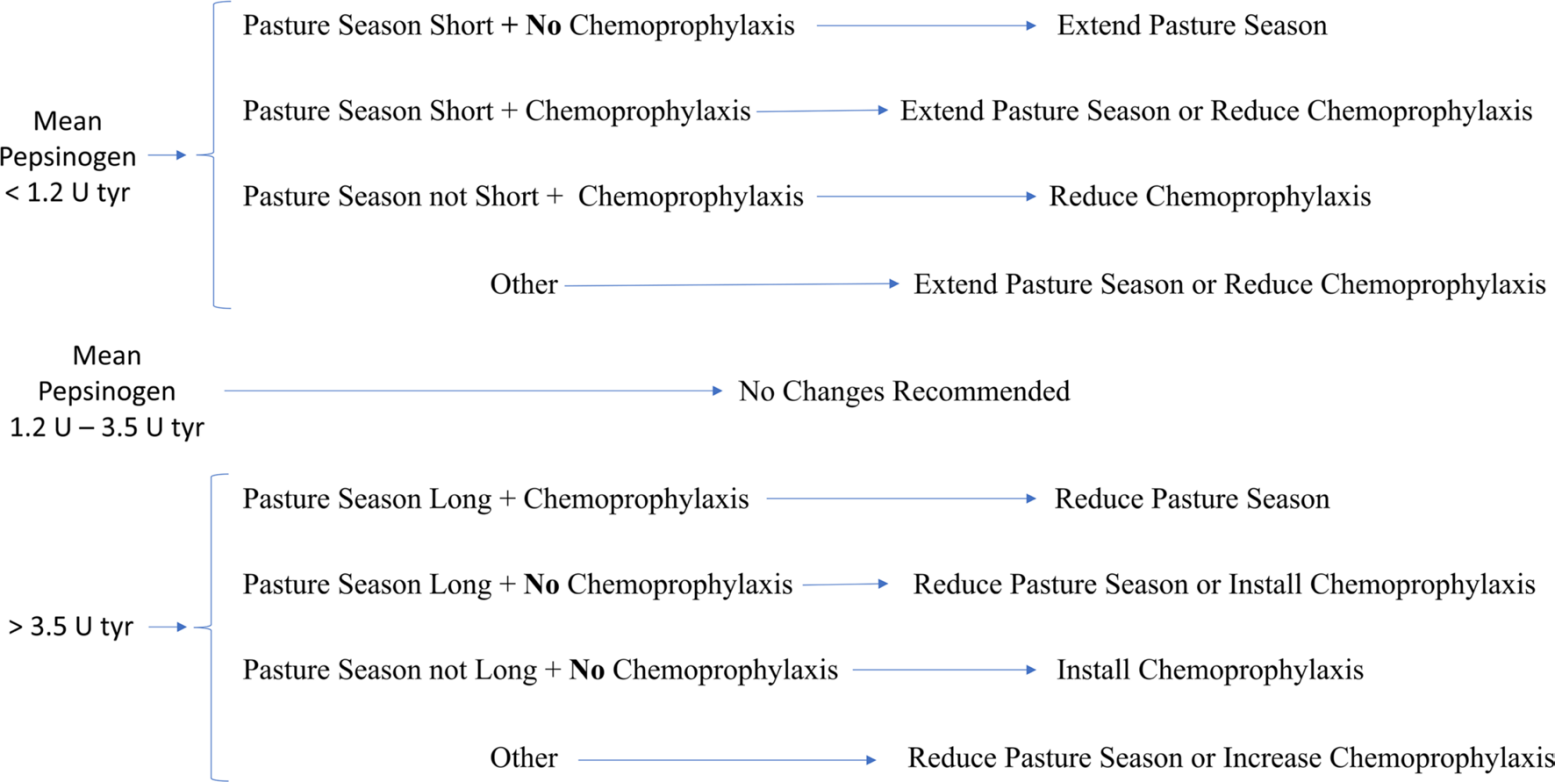
Flow chart used to provide worm control advice in the first-season grazing calves. A pasture season is considered short when ≤ 3 months and long when ≥ 6 months. “Other” means that no classification could be made based on the lack of clarity of available information [74]

Guidance on the practical implementation of pepsinogen measurements on farms has been previously provided [74]. Broadly, it is recommended that six to seven animals out of a group of up to 40 animals should be tested at stabling and that information on the length of the grazing season and intensity of the chemical treatment (chemoprophylaxis) should be collected. These three factors can be used to assess exposure as a proxy for both the effectiveness of control and the level of *O.* *ostertagi* immunity acquired. By following the flow chart in Fig. 3 [74], a practical parasite control recommendation for the following year can be developed.

It should be noted that despite the practical value of this technique in the field, it is mostly used by researchers, mainly due to its cost and the invasive sampling obligation.

## *Fasciola hepatica*

The definitive diagnostic test for *F.* *hepatica* is liver necropsy, which provides a highly accurate diagnosis of fasciolosis when the bile ducts are carefully dissected [75]. Clearly, this is not a practical option as a herd or flock management tool, as it can only be carried out postmortem [76].

The most frequently used ante-mortem diagnostic test is the detection of eggs in feces by sedimentation or flotation techniques, expressed as the FEC [77]. Unpublished data by the Animal and Plant Health Agency (APHA) of the UK show that the sedimentation technique is more sensitive than flotation in zinc sulphate (note: a technique that consists of sedimentation and then flotation was not assessed). The sedimentation technique also allows easy differentiation of the eggs of liver flukes from those of rumen flukes. Despite the benefits of fecal exams for *Fasciola* egg detection, the *Fasciola* pre-patent period is 8–10 weeks depending on the host species; hence, egg counts are only useful from approximately 8 weeks post-infection onwards. In addition, other factors, such as host age, fecal water content and the number of aliquots tested per sample, can all affect the sensitivity of the FEC [78]. False positives or false negatives may occur due to the retention of eggs in the gall bladder for at least 2 weeks after successful treatment [79]. It is worth noting that repeated testing or analysis of > 30 g of feces can increase the detection rate to up to 90% [75, 80]. FEC can be a poor indicator of infection when the parasite burden is low or when nonreproducing immature stages are migrating [81, 82].

Coprological sedimentation/flotation methods are well established in routine diagnostic laboratories, and methods such as FLOTAC [33] and Flukefinder® [83] can also be used to detect *F.* *hepatica* eggs. Flukefinder® is a commercially available egg detection device based on a modified sedimentation and a fine filtration technique. It is commonly used in veterinary diagnostic laboratories across Europe and North America [84]. Flukefinder® is more effective than the simple sedimentation method at retrieving fluke eggs in cattle and sheep [83].

It has been suggested that animals with as few as 1–10 parasites grow at a slower rate than uninfected animals [85]. If this is the case, diagnostic tools with a low detection limit are very important. It may also be advisable to detect low fluke burdens in sheep as well because of the high sensitivity of sheep to this parasite.

As an alternative to FECs, liver fluke-specific ELISAs have been developed and are being routinely used in cattle and sheep. *Fasciola* *hepatica*–ELISAs are adaptable tests that detect specific antigens in feces or antibodies in pooled or individual milk or sera. The most damaging stage of this infection in the final host occurs during the migration of immature stages, and the failure of FECs as a tool to diagnose immature migrating stages of the liver fluke in the final host is a major disadvantage of this method. In comparison, a major advantage of the ELISA tests is the detection of early infection. In addition, ELISA techniques have demonstrated improved sensitivity of diagnosis over coprological methods [86, 87].

The detection of *F.* *hepatica* antigens in feces has been shown to have high sensitivity and specificity [86, 88]. The coproantigen ELISA detects excretory/secretory antigens secreted by live adult and late-immature *Fasciola* into feces. The MM3—Copro ELISA (Bio-X Diagnostics, Rochefort, Belgium) has been shown to detect 100% of sheep with one fluke and 100% of cattle with two flukes [89]. The first detection of *F.* *hepatica*-specific coproantigens by the MM3 capture ELISA preceded the first detection in the egg count by 1 to 5 weeks. In sheep that were experimentally infected and then treated with flukicide, coproantigens became undetectable from 1 to 3e weeks post-treatment. The MM3—Copro ELISA had no cross reaction when tested in co-infections with paramphistome, coccidian and/or GINs [90, 91]. Therefore, this test could be used instead of fecal examination for fluke eggs (as it is potentially a cheaper test) or to evaluate flukicide efficacy.

Another available ELISA kit for *F.* *hepatica* diagnosis is the SVANOVIR® *F.* *hepatica*-Ab (Svanova-INDICAL Sweden AB, Uppsala, Sweden). The results from this diagnostic tool have been proven to be strongly correlated with infection (number of flukes in the liver), antibody levels to *F.* *hepatica* and loss of milk yield or carcass weight [92, 93]. SVANOVIR® *F.* *hepatica*-Ab has been validated in dairy and beef cattle using milk and serum/meat juice samples, respectively, thus enabling the monitoring of fasciolosis at several different stages of the production chain, including on farms, at dairies and at slaughter. In this context, it should be noted that fluke antibodies can persist for several months after successful treatment.

Blood ELISA tests for *F.* *hepatica-*Ab in areas where *F.* *hepatica* is unusual can be used to diagnose infection. In western Europe, this test can be used to indicate first infection in home-bred first-year grazing animals to help identify the timing of the increase in metacercariae in the autumn. This allows animals to be more accurately treated for acute fasciolosis.

Several other ELISA kits have been developed for the detection of *F.* *hepatica* infection in bulk tank milk samples. They can also be used to assess the treatment response in dairy herds. It is important to consider the treatment measures applied, age and milking period of the herd before interpreting bulk tank milk ELISA results [92].

Table 7 lists the liver fluke diagnostic techniques and their characteristics and provides guidance on the situations that each of them can be used [94].

**Table 7:** Available liver fluke diagnostic techniques, when to use them and their characteristics (valid for western Europe)

Test	When to use it	Sampling	Diagnostic value	Drawbacks
Blood antibody ELISA	From approx. 3–4 weeks post-infection	Regular blood sampling. Use first-season grazing animals (lambs and/or calves) as “sentinels” and 10 animals per risk group (consider on-farm risks, such as grazing)	Measure of acute disease risk. Increasing antibody levels identify when active infection is occurring for targeted treatment	Careful interpretation of test results is required to avoid premature treatment. Test results for sentinel animals indicate risk status only for their group. Antibody levels can remain high even after successful treatment and in previously exposed older animals
Coproantigen ELISA	From approx. 8–10 weeks post-infection	Dung, individual (avoid using pooled if possible)	Mid- to late-stage infection	Low sensitivity in cattle and in pooled samples. If result is negative, advise re-test in approx. 4 weeks
Milk ELISA	From approx. 2–4 weeks post-infection	Bulk milk tank	Monitoring the parasite-infection status of a herd Test results are associated with parasite-induced production losses	Antibodies can persist until 8 months after effective treatment
Fluke egg counts	From approx. 10–11 weeks post-infection	Dung, individual and pooled	Definitive diagnosis when adult parasites present	Test sensitivity may be low, especially in cattle. If result is negative, advise re-test in 4–8 weeks
Post-mortem	From approx. 2 weeks post-infection	Fallen stock	Definitive diagnosis (all stages of infection)	Abattoir returns are useful, but should not be considered equivalent to veterinary post-mortem in terms of reliability

*ELISA* Enzyme-linked immunosorbent assay

Adapted from: Liver fluke. A guide to test-based control [94]

## Lungworm

Persistent cough is the most common clinical sign of dictyocaulosis in cattle. Particularly in humid temperate regions, disease should be suspected in any coughing cattle with access to pasture, usually from the middle to the end of the grazing period. The clinical signs of coughing, reduced exercise tolerance and a heavy, fast respiration rate are easiest to observe when cows are brought in for milking or moved between paddocks. Sudden death may also be observed, especially in the case of reinfection syndrome. The typical ‘lungworm stance,’ with cattle standing with their head extended and tongue protruding, is not seen in every case but should be looked for. More subtle signs of weight loss and reduced milk yield may be the only clinical feature.

Table 8 shows several infectious organisms giving similar clinical signs, and concomitant infections are not rare. This can make it difficult to estimate the relative importance of lungworm when disease is observed. At pasture, lungworm should be considered a likely ‘stressor,’ enabling disease by infectious organisms which, on their own, would not develop. For example, lungworm infections may cause recrudescence of latently present infectious bovine rhinotracheitis (IBR) virus, with regular coughing creating a vehicle for IBR spread [95].

**Table 8:** Agents to be considered in the differential diagnosis of lungworm disease

Disease type	Pathogen
Viral pathogens	Infectious bovine rhinotracheitis Respiratory syncytial virus—can be primary cause in dairy cattle Parainfluenza 3 Bovine viral diarrhoea virus—primary cause and/or immune suppression Coronavirus—often present but pathogenicity unknown
Bacterial pathogens	*Mannheimia* *haemolytica* *Pasteurella* *multocida* *Haemophilus* *somnus* *Mycoplasma* *bovis*
Others	Farmers’ lung (i.e. hypersensitivity to spores of *Micropolyspora* *faeni*) Dusty feed rhinotracheitis (i.e. feeding fine-particled feed in a badly ventilated parlour) *Chlamydia* *pecorum*—detected by PCR, but pathogenicity unknown *Anaplasma* *phagocytophilum*

Clinical signs usually start after the second week of infection; however, both the Baermann and ELISA tests, which reveal the presence of adult worms, will only be positive from post-infection days 23 to 28 onwards. This ‘diagnostic gap’ presents a challenge, especially when very few animals are clinically affected. Importantly, cattle that are fully or partially immune, including those suffering ‘reinfection syndrome,’ will normally harbor immature worm burdens and then not test positive in either test. During the prepatent period, bronchoalveolar lavages can provide invaluable information, but these are perceived to be time-consuming and therefore are arguably underused as a diagnostic tool. The diagnosis is easily reached during postmortem examinations. The ELISA diagnostic kit for lungworm is beyond the scope of the present review due to its restricted geographical availability (only in UK and The Netherlands).

## Baermann test

The Baermann test presents a high sensitivity in calves when at least 30 g of feces is examined [96]. Individual samples from several animals showing clinical signs should be analyzed. If an average herd size of 73 animals (19 heifers and 54 cows) is considered, nine heifers and 15 cows (second lactation or later) should be individually tested for at least one positive result [97]. To lower the chances of a false-negative test, it is crucial that samples are refrigerated and processed rapidly. Even when kept in the fridge, 20% of first-stage (L1) larvae are likely to die within 24 h of sampling. At room temperature, 60% and 80% of L1 larvae will have died after 24 and 48 h, respectively [98]. False positive or false negative results can occur if samples are left for a sufficient length of time for gastrointestinal eggs to have hatched into L1 larvae and/or lungworm L1 to have died.

## Conclusions

Diagnostics is one of the pillars of a modern parasite control program. Parasite control best practices can be summarized as treating the right animal with the right product, with the right dose and at the right time, and last, but not least with proper management of the pasture. This statement might be simple, but the implementation of parasite control best practices remains a challenge for most of veterinarians/producers around the world. In this review, we first identified the right animals to be treated. Unfortunately, a convenient (pen-sided, easy-to-use and low processing time) low-cost diagnostic technique with a low detection limit, high accuracy and high precision is not yet available. Nevertheless, by understanding which diagnostic tools are accessible and knowing their advantages and limitations, it is possible to choose the most suitable method for each production operation. While the results of some diagnostic techniques might not be straightforward, when used together with other farm data, such as parasite control history, parasite epidemiology, husbandry practices and climate, they are the foundation of an evidence-based discussion between veterinarians and producers on sustainable parasite control. Parasites have developed resistance to most of the anthelmintic drug classes currently available on the market, and it is unlikely that the development of innovative products (containing active pharmaceutical ingredients with new modes of action) will outpace the advance of resistance. This is why all ruminant parasitology stakeholders (farmers, veterinarians, researchers, regulators and the animal health industry) should be working on practices that enable the employment of best practices for sustainable parasite control, and proper parasitological diagnosis is the first step towards this objective.

## Data Availability

Not applicable.

## Abbreviations

EPG: Eggs per gram (of feces)
ELISA: Enzyme-linked immunosorbent assay
FEC: Fecal egg count
GIN: Gastrointestinal nematode
TST: Targeted selective treatment
